# Bilateral Laparoscopic Gonadectomy in a Patient With Complete Androgen Insensitivity Syndrome and Bilateral Sertoli-Leydig Cell Tumor: A Case Report and Brief Review of the Literature

**DOI:** 10.5812/numonthly.15278

**Published:** 2014-04-21

**Authors:** Mohammad Asl Zare, Mahmood Reza Kalantari, Amir Abbas Asadpour, Ali Kamalati

**Affiliations:** 1 Department of Urology, Faculty of Medicine, Mashhad University of Medical Sciences, Mashhad, IR Iran; 2 Department of Pathology, Faculty of Medicine, Mashhad University of Medical Sciences, Mashhad, IR Iran; 3 Department of Urology, Faculty of Medicine, Kerman University of Medical Sciences, Kerman, IR Iran

**Keywords:** Androgen-Insensitivity Syndrome, Sertoli-Leydig Cell Tumor, Laparoscopy

## Abstract

**Introduction:**

Complete androgen insensitivity syndrome (previously called testicular feminization) is specified by a 46 XY karyotype and negative sex chromatin, bilateral undescended testes, female genitalia appearance, and lack of mullerian derivatives.

**Case Presentation:**

A 28-year-old woman with complete (severe) androgen resistance underwent prophylactic laparoscopic bilateral gonadectomy because of the eventually increased risk of gonadal malignancy. Although the gonads appeared grossly normal, microscopic examination revealed bilateral well differentiated sertoli–leydig cell tumor (SLCT).

**Discussion:**

Our Medline search revealed that this is the first reported case of bilateral sertoli–leydig cell tumor (SLCT) in androgen insensitivity syndrome.

## 1. Introduction

Complete androgen insensitivity syndrome (AIS - previously called testicular feminization) is specified by a 46 XY karyotype and negative sex chromatin, bilateral undescended testes, female genitalia appearance, and lack of mullerian derivatives. The patient is phenotypically female without uterus and a blind shortened vagina (1). This syndrome is often diagnosed during puberty while the patient is being evaluated due to primary amenorrhea (2).

Complete AIS is accompanied with abnormal testicular development and increase risk of germ cell malignancy beginning after puberty. For these reason, prophylactic gonadectomy is advised in the postpubertal period to avoid potential malignant transformation (2). We present the first documented case of bilateral sertoli–leydig cell tumor (SLCT) in a patient with complete (severe) AIS.

## 2. Case Presentation

A 28-year-old nulligravida was referred for consultation and laparoscopic bilateral gonadectomy after diagnosis of complete androgen resistance. The patient complained of primary amenorrhea but she did not have inguinal hernia or labial mass. She had female external genitalia. In the physical examination, development of her breasts was normal (tanner stage IV), but axillary hair was scarce (tanner stage III). Abdominal examination was normal. No inguinal or labial mass was palpated. In gynaecological examination, the vulva and perineum appeared normal. She had a blind-ending vaginal pouch of approximately 6 cm depth and the cervix was invisible. No adnexal masses were palpated.

In pelvic ultrasonography, the uterus was absent and an ovoid heterogenous mass (38 × 23 mm in size) with a 13 mm nodule in the right pelvic sidewall had been reported. Abdominal ultrasonography did not reveal any para-aortic lymphadenopathy. Pelvic ultrasonography failed to identify the left gonad. Blood tests for hormones showed Follicle-stimulating hormone and luteinizing hormone levels were 7 mIU/mL and 32 mIU/mL, respectively and serum testosterone was 3.1 ng/mL, all of which were within the normal ranges. Cytogenetic analysis showed a male karyotype (46, XY).

**Figure 1.**
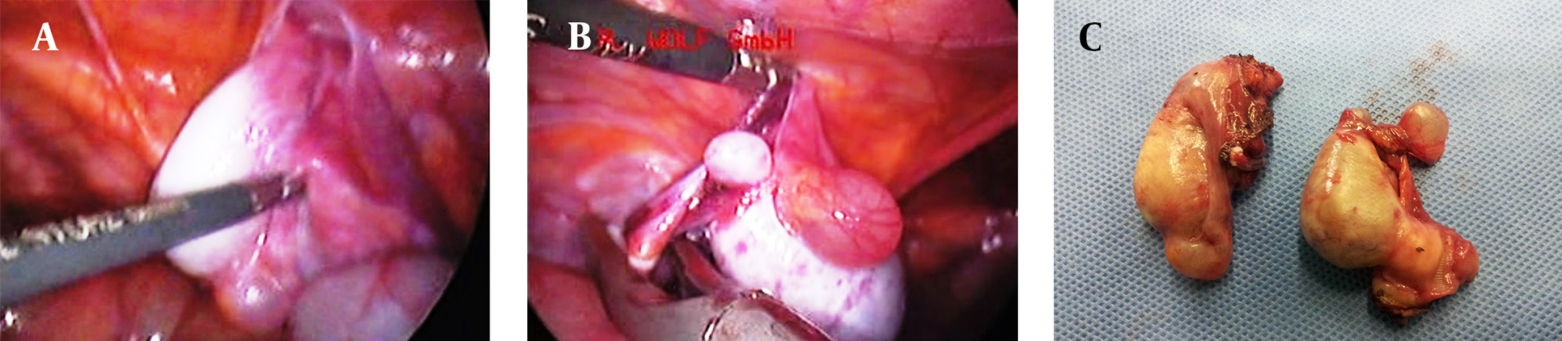
A. Laparoscopic View of the Left Gonad; B. The Right Gonad; C. Gross Image of the Removed Gonads

The patient underwent laparoscopic gonadectomy under general anesthesia in the supine position. Visualization of pelvic contents revealed absence of uterus and fallopian tubes. There was not any evidence of abdominal or pelvic lymphadenopathy. Laparoscopic study demonstrated a 4 × 2.5 cm gray-purple colored ovoid mass with 1 cm cystic mass near the right internal inguinal ring. The left gonad (4.5 × 2 cm) was hidden behind the bowels in the left pelvic sidewall. The gonad and the attached structure were retracted medially. The posterior peritoneum and gonadal vessels were incised after coagulation and ligation, and the gonads were removed from the pelvic sidewalls (Figure 1).

**Figure 2.**
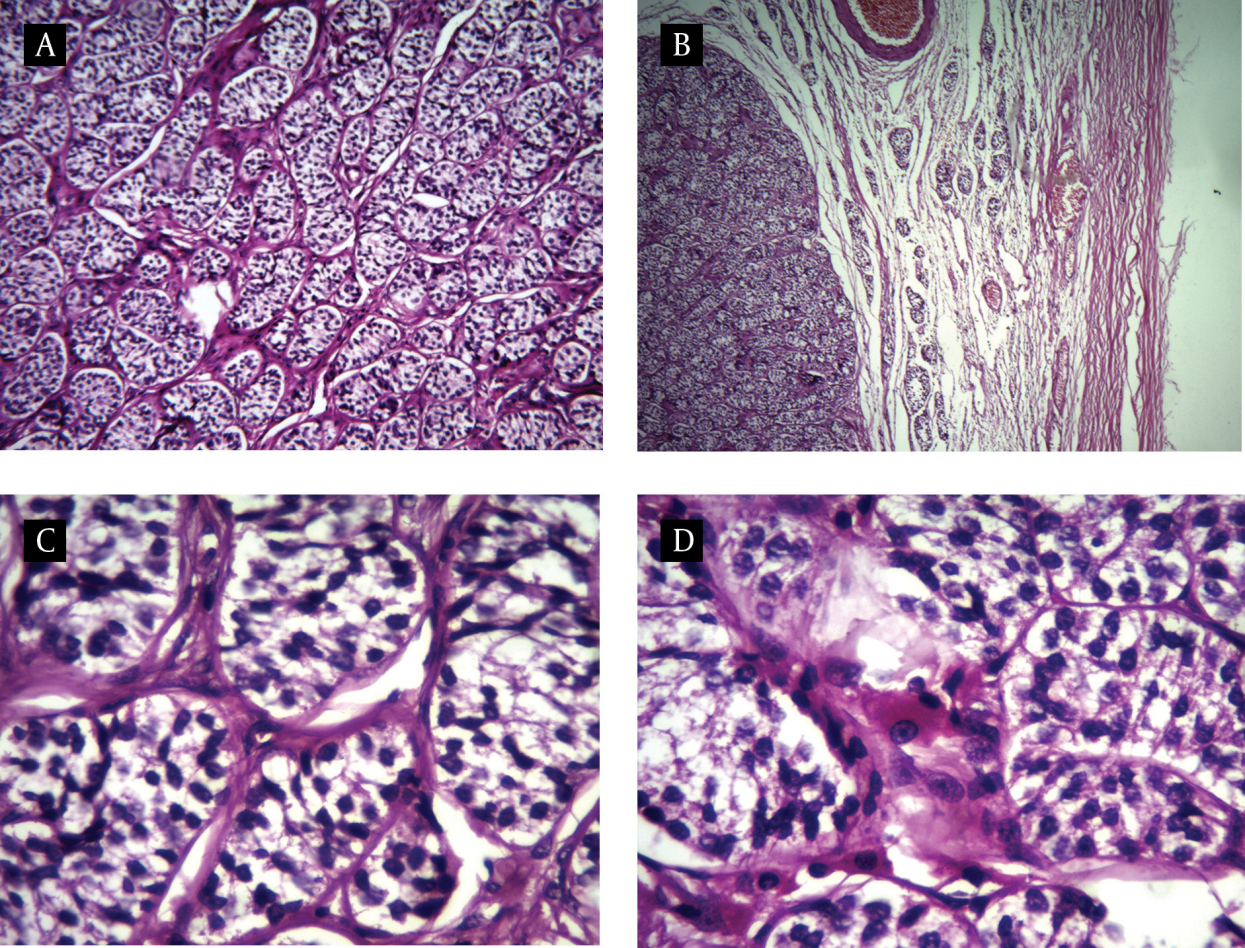
Well-differentiated Sertoli-Leydig Cell Tumor: A. The Tumor Is Well Circumscribed; B. Tumor Growth in Aluminal Tubular Pattern; C. Tumoral Cells Reveal Dark Round to Oval Nuclei and Clear Cytoplasm; D. Inervening Stroma Contain of Large Polygonal Cells Reminiscence to Leydig Cells.

Macroscopically, the specimen consist of two ovoid gray mass which one of them contained two well-delineated, homogenous yellow-tan nodules measuring 1.5 and 1 cm on cut surface, respectively. Another mass on cut surface contained one 2.3 cm nodule similar to above description. Histopathological examination of the gonads in non-neoplastic region showed prepubertal immature tubules with lined sertoli cells. Microscopic examination of delineated nodules revealed a luminal-tubular pattern populated by monomorphus cells with dark round to oval nuclei reminiscence of immature sertoli cells. Intervening stroma was scant and focally contained clusters of polygonal cells with abundant eosinophilic or vacuolated cytoplasm and round nucleus with prominent nucleoli. Mitotic activity was not prominent and necrosis was not seen (Figure 2). These findings were consistent with bilateral well differentiated sertoli–leydig cell tumor (SLCT). Our patient is being followed up regularly and she has no complain after a year.

## 3. Discussion

AIS is the most common type of male pseudohermafroditism characterized by absence of androgen receptor (AR) activity due to a mutation at Xq11–q12 localization on the androgen receptor gene (3-5). It is known as an X-linked recessive disease, but up to 30% of mutations may be presented as sporadic de novo mutations (6). The incidence of this condition is 1 in 99,000 to 1 in 20,000 female births (7-9). These patients may be seen with a spectrum of phenotypic disorders that varies from pure female complete androgen insensitivity syndrome (CAIS), ambiguous genitalia partial androgen insensitivity (PAIS) and to phenotypically infertile male minimal androgen insensitivity form (MAIS) (2).

The complete form is 10 times more common than the incomplete form and the initial presentation is primary amenorrhea in adolescents with female phenotype, lack of pubic hair, or inguinal hernia containing testis and characterized by a 46 XY genotype and normal male androgen and gonadotropin value. Pelvic ultrasonography usually shows absence of mullerian derivates and vaginal examination reveals a blind-ending vagina without a cervix. Notably, on physical examination, all of these characteristics were present in our patient (2). In children or infants with a female phenotype, androgen insensitivity syndrome may present as inguinal hernia or mass and ambiguous genitalia. About 50% of patients with complete (severe) AIS have an inguinal hernia. Conversely, 1-2% of apparently female infants with inguinal hernia are diagnosed to have a 46 XY karyotype and complete AIS. It may be found during workup for lower abdominal pain (10), abdominal mass (11), painful intercourse, or diagnosed incidentally on imaging investigations (12). The testes may be found in the abdomen, inguinal canal, or labia. Abnormalities of testicular development and risk of gonad malignancy increase after puberty.

Testis tumor developing risk is thought to be 3.6% by 25 years and 33% by 50 years (11, 13). In a case series of 43 patients with AIS by Rutgers and Scully, 63% hamartomas, 23% sertoli cell adenomas and 9% malignant tumors including two seminomas, one intratubular germ cell neoplasm with early stromal invasion and a malignant sex cord tumor were reported (13). We reviewed the previous articles on gonadal tumors in CAIS patients (Table 1). Most cases are unilateral sertoli adenoma or tumor. There are only two reported cases of bilateral gonadal tumors (10, 14). Also, there are only two cases of sertoli-leydig cell tumor which both were unilateral (15, 16).

**Table 1.** Reported Cases of Sex Cord Stromal Tumors in Patients With Complete Androgen Insensitivity Syndrome

Tumor Type	Age, y	Tumor Size, cm	Localization	Reference
**Sertoli cell adenoma **	21	7.5 × 3	right gonad	O’Connell et al. (17)
**Sertoli cell adenoma**	82	24 × 19	left gonad	Damjanow et al. (18)
**Sertoli cell adenoma **	20	5 × 2.5	left gonad	Ramaswamy et al. (19)
**Sertoli cell adenoma (10 cases)**	17-53	0.5 to 14	left gonad	Rutgers and Scully (13)
**Large cell calcifying sertoli cell tumor / sex cord tumor with annular tubules (2 caces)**	18 and 20	1 to 8	left gonad	Rutgers and Scully (13)
**Sertoli cell tumor**	26	26 × 17	bilateral	Wysocka et al. (14)
**Sertoli cell adenoma**	22	2	left gonad	Ko et al. (20)
**Sertoli cell tumor and adenocarcinoma of tunica vaginalis testis**	72	35 × 25		Fleckenstein et al. (21)
**Sertoli cell adenoma and serous cyst**	30		bilateral	Baksu et al. (10)
**Malignant leydig cell tumor**	73	10.5 × 9.5	left gonad	Iwamoto et al. (11)
**Sertoli-leydig cell tumor and ureteral transitional cell carcinoma**	57	2 × 1.8	right gonad	Choi et al. (22)
**Sertoli cell adenoma**	53	14 × 10 × 10	left gonad	Scully et al. (23)
**Sertoli cell tumor**	60	large abdominal tumor		Knoke et al. (24)
**Sertoli cell adenoma**	67	15 × 14 × 10	left gonad	Lentz et al. (25)
**Sertoli cell adenoma**	81	16 × 11 × 6	left gonad	Detre and Bujdoso (26)
**Large cell calcifying sertoli cell tumor/sex cord tumor with annular tubules (2 cases)**	19	8 × 8 × 4.5	right gonad	Ramaswamy et al. (19)
**Sertoli-leydig cell tumor**				Jarzabec et al. (15)
**Sertoli-Leydig cell tumor**	29		left gonad	Ozulker et al. (16)
**Sertoli cell tumor and ITGCN**	15		separate gonads	Lin et al. (27)
**Sertoli-leydig cell tumor**	28	4 × 2.5	Bilateral	present case

In AIS pateints, prophylactic gonadectomy is advised in the postpubertal period due to avoid potential malignant transformation of the gonads (2, 28). Gonadectomy is recommended during the postpubertal period to help the development of feminization during puberty when the malignant changes in germ cells are relatively late and rare (13).

Sertoli-leydig cell tumor is a rare sex cord stromal neoplasm that account for less than 1% of ovarian tumors, occurring often in young adults (29, 30). It is also seldom developed among the patients with AIS and there is only two unilateral cases reported in the literature (15, 16). According to the amount of tubular differentiation of the sertoli cells and quantity of the primitive gonadal stroma, sertoli-leydig cell tumor is divided into well, intermediate and poorly differentiated types. Our patient had a well-differentiated form of SLCT, which is the most infrequently type seen with the good prognosis. To our knowledge, this is the first documented case report of bilateral benign sertoli-leydig cell tumor (SLCT) in androgen insensitivity syndrome.
